# Supplementary material for: The Small RNA Universe of Capitella teleta
Source: Front Mol Biosci. 2022 Feb 25;9:802814. doi: 10.3389/fmolb.2022.802814 (PMC8915122; doi:10.3389/fmolb.2022.802814)
Supplement: Supplementary file 1 [file DataSheet1.ZIP › Supplement/known/CAPTEscaffold_12_1215.pdf]

Provisional ID	:	CAPTEscaffold_12_1215
Score total	:	6821.8
Score for star read(s)	:	3.9
Score for read counts	:	6815.5
Score for mfe	:	1.5
Score for randfold	:	1.6
Score for cons. seed	:	-0.6
Total read count	:	13380
Mature read count	:	13330
Loop read count	:	0
Star read count	:	50

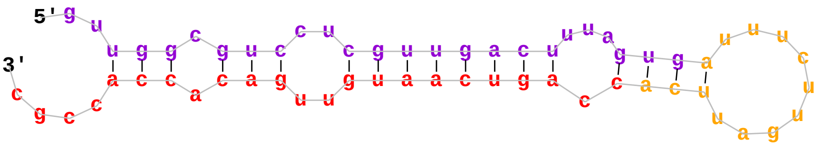

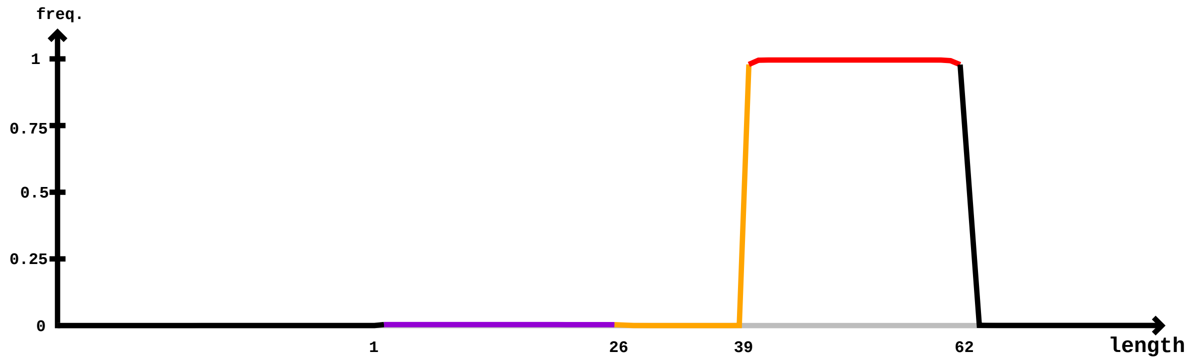


```
                                               Star                                   Mature
5'- ugacuacuugguacugcaugcucauagcaagcguuggcguccucguugacuuuagugauuucuugauucaccagucaauguugacaccaccgccugacuugagccaauucauc   -3'   obs
    ugacuacuugguacugcaugcucauagcaagcguuggcguccucguugacuuuagugauuucuugauucaccagucaauguugacaccaccgccugacuugagccaauucauc         exp
    ugacuacuugguacugcaugcucauagcaagcguuggcguccucguugacuuuagugauuucuugauucaccagucaauguugacaccaccgccugacuugagccaauucauc         known
    ...................(((((.((((.(((.(((.(((..((((((((...((((.........)))).))))))))..))).))).))).)).))))))).........    reads     mm     sample
    .............cugcaugcucauagcaagc.................................................................................    2         0      seq
    ................................guuggcguccucguugacu..............................................................    5         0      seq
    ................................guuggcguccucguugacuuuagu.........................................................    1         0      seq
    ................................guuggcguccucguugacuuuagug........................................................    33        0      seq
    ................................guuggcguccucguugacuuuagugU.......................................................    8         1      seq
    ................................guuggcguccucguugacuuuagugUu......................................................    1         1      seq
    ................................guuggcguccucguugacuuuagugUuu.....................................................    2         1      seq
    .....................................................................accagucaauguugacaccaccgc....................    3         0      seq
    ......................................................................ccagucaauguugacaccac.......................    1         0      seq
    ......................................................................ccagucaauguugacaccacc......................    31        0      seq
    ......................................................................ccagucaauguugacaccaccU.....................    1         1      seq
    ......................................................................ccagucaauguugacacUaccg.....................    1         1      seq
    ......................................................................ccagucaauguugacaccaccg.....................    195       0      seq
    ......................................................................ccagucaauguuAacaccaccg.....................    1         1      seq
    ......................................................................Acagucaauguugacaccaccgc....................    2         1      seq
    ......................................................................ccagucaaugGugacaccaccgc....................    1         1      seq
    ......................................................................ccagucaauguugGcaccaccgc....................    6         1      seq
    ......................................................................ccagucUauguugacaccaccgc....................    1         1      seq
    ......................................................................ccaUucaauguugacaccaccgc....................    1         1      seq
    ......................................................................ccagucaauguugCcaccaccgc....................    8         1      seq
    ......................................................................Ncagucaauguugacaccaccgc....................    5         1      seq
    ......................................................................ccagucaauguCgacaccaccgc....................    2         1      seq
    ......................................................................ccagucaaugAugacaccaccgc....................    6         1      seq
    ......................................................................cAagucaauguugacaccaccgc....................    5         1      seq
    ......................................................................ccagAcaauguugacaccaccgc....................    8         1      seq
    ......................................................................ccagucaauguugacCccaccgc....................    1         1      seq
    ......................................................................ccagucaauguugacacAaccgc....................    11        1      seq
    ......................................................................cUagucaauguugacaccaccgc....................    11        1      seq
    ......................................................................ccagucaauguuAacaccaccgc....................    5         1      seq
    ......................................................................ccagucaauguugacaccaUcgc....................    2         1      seq
    ......................................................................ccagucaauguugacaccaccCc....................    1         1      seq
    ......................................................................ccagucaauguugaGaccaccgc....................    1         1      seq
```

Star Mature

```
ugacuacuugguacugcaugcucauagcaagcguuggcguccucguugacuuuagugauuucuugauucaccagucaauguugacaccaccgccugacuugagccaauucauc
......................................................................ccagucaauguugacaccaccAc....................  8      1  seq
......................................................................ccagCcaauguugacaccaccgc....................  1      1  seq
......................................................................ccaguUaauguugacaccaccgc....................  4      1  seq
......................................................................ccagucaauguugacaccaccgc....................  12196  0  seq
......................................................................ccaguAaauguugacaccaccgc....................  2      1  seq
......................................................................ccagucaauguugUcaccaccgc....................  1      1  seq
......................................................................cNagucaauguugacaccaccgc....................  1      1  seq
......................................................................ccagucaaGguugacaccaccgc....................  1      1  seq
......................................................................ccagucaauguugacaccaccgA....................  4      1  seq
......................................................................ccagucaauguugacUccaccgc....................  2      1  seq
......................................................................ccagucaauguAgacaccaccgc....................  8      1  seq
......................................................................ccagucaaugCugacaccaccgc....................  1      1  seq
......................................................................ccagucaaAguugacaccaccgc....................  4      1  seq
......................................................................ccagucaauguugacaGcaccgc....................  1      1  seq
......................................................................ccUgucaauguugacaccaccgc....................  4      1  seq
......................................................................ccagucaauguugacaccGccgc....................  2      1  seq
......................................................................ccagucaauguugaAaccaccgc....................  3      1  seq
......................................................................ccagucaauguugacacUaccgc....................  4      1  seq
......................................................................ccagucaauguugacaccacUgc....................  5      1  seq
......................................................................ccagucGauguugacaccaccgc....................  1      1  seq
......................................................................ccagucaauguGgacaccaccgc....................  1      1  seq
......................................................................ccagucaauguugacaccacAgc....................  11     1  seq
......................................................................ccagucaauguuCacaccaccgc....................  1      1  seq
......................................................................ccGgucaauguugacaccaccgc....................  1      1  seq
......................................................................ccagucaaCguugacaccaccgc....................  1      1  seq
......................................................................ccagucaauguugacaccaAcgc....................  6      1  seq
......................................................................ccagucaauguugacaccaccgG....................  1      1  seq
......................................................................ccagucaauguugaUaccaccgc....................  4      1  seq
......................................................................Ucagucaauguugacaccaccgc....................  12     1  seq
......................................................................ccagucaauguugacGccaccgc....................  4      1  seq
......................................................................ccagucaauguugacaccaccgU....................  1      1  seq
......................................................................ccagucaauguugacaccaccgcU...................  464    1  seq
......................................................................ccagucaauguugacaccaccgcG...................  2      1  seq
......................................................................ccagucaauguugacaccaccgcA...................  33     1  seq
......................................................................ccagucaauguugacaccaccgcUu..................  4      1  seq
......................................................................ccagucaauguugacaccaccgccuU.................  1      1  seq
.......................................................................cagucaauguugacaccaccgc....................  206    0  seq
.......................................................................cagucaauguugacaccaccgcU...................  5      1  seq
........................................................................agucaauguugacaccaccgc....................  9      0  seq
.............................................................................................cugacuugagccaauucau.  1      0  seq
.............................................................................................cugacuugagccaauucauc  5      0  seq
```
